# A Neighborhood Approach for Using Remotely Sensed Data to Estimate Current Ranges for Conservation Assessments

**DOI:** 10.1002/ece3.71631

**Published:** 2025-06-27

**Authors:** Bethany A. Johnson, Gonzalo E. Pinilla‐Buitrago, Robert P. Anderson

**Affiliations:** ^1^ Department of Biology, City College of New York City University of New York New York New York USA; ^2^ Center for Biodiversity and Conservation American Museum of Natural History New York New York USA; ^3^ Ph.D. Program in Biology, Graduate Center City University of New York New York New York USA; ^4^ Division of Vertebrate Zoology (Mammalogy) American Museum of Natural History New York New York USA

**Keywords:** biogeography, conservation, ecological niche modeling, remote sensing, species distribution modeling, uncertainty

## Abstract

Species distribution modeling can be used to predict environmental suitability, and removing areas currently lacking appropriate vegetation can refine range estimates for conservation assessments. However, the uncertainty around geographic coordinates can exceed the fine resolution of remotely sensed habitat data. Here, we present a novel methodological approach to reflect this reality by processing habitat data to maintain its fine resolution, but with new values characterizing a larger surrounding area (the “neighborhood”). We implement its use for a forest‐dwelling species (*Handleyomys chapmani*) considered threatened by the IUCN. We determined deforestation tolerance threshold values by matching occurrence records with forest cover data using two methods: (1) extracting the exact pixel value where a record fell; and (2) using the neighborhood value (more likely to characterize conditions within the radius of actual sampling). We removed regions below these thresholds from the climatic suitability prediction, identifying areas of inferred habitat loss. We calculated Extent of Occurrence (EOO) and Area of Occupancy (AOO), two metrics used by the IUCN for threat level categorization. The values estimated here suggest removing the species from threatened categories. However, the results highlight spatial patterns of loss throughout the range not reflected in these metrics, illustrating drawbacks of EOO and showing how localized losses largely disappeared when resampling to the 2 × 2 km grid required for AOO. The neighborhood approach can be applied to various data sources (NDVI, soils, marine, etc.) to calculate trends over time and should prove useful to many terrestrial and aquatic species. It is particularly useful for species having high coordinate uncertainty in regions of low spatial autocorrelation (where small georeferencing errors can lead to great differences in habitat, misguiding conservation assessments used in policy decisions). More generally, this study illustrates and enhances the practicality of using habitat‐refined distribution maps for biogeography and conservation.

## Graphical abstract

We created a neighborhood approach for processing habitat data used in conjunction with species distribution modeling to improve range estimates for conservation assessments.

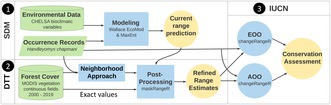

## 1 Introduction

Accurate estimates of the geographic areas suitable for a species are important for their use in biodiversity assessments and climate change research (Peterson et al. 2011; Araújo et al. 2019). Quantification of a species' range can be used to calculate conservation metrics, such as those used by the International Union for Conservation of Nature (IUCN) for threat level assessments (IUCN 2022), which rely on accurate estimates to categorize threat severity. Two such IUCN metrics relevant for assessing population size reduction for Criterion A and decline of geographic range for Criterion B are the Extent of Occurrence (EOO) and Area of Occupancy (AOO). EOO measures the spatial spread of inferred or projected sites and is commonly calculated from a minimum convex polygon around known localities (IUCN 2022) but also can be based on the analogous polygon around a map of suitable habitat in the species' range (Kass et al. 2021a). AOO is a subset of the EOO that measures presence within available habitat at a standardized 2 × 2 km spatial grid (IUCN 2022). They each quantify aspects of a species' range, but since AOO focuses on occupied suitable habitat, it can highlight vulnerabilities to habitat loss or degradation, especially for species in fragmented landscapes. Whereas EOO estimates are generally rather stable via different methodological options, several approaches for calculating AOO bracket a vast range from a lower bound of underestimation to an upper bound of overestimation (Anderson 2022). At one extreme, summing the total area of occupied cells (where documented occurrence localities fall) within the 2 × 2 km grid gives the lower bound estimates of AOO, but this typically leads to strongly biased underestimates since most cells lack any sampling. On the other end, an upper bound estimate sums the 2 × 2 km grid cells with suitable habitat within the range (e.g., area of habitat, AOH; Brooks et al. 2019), assuming the species occupies all of them, which typically yields biased overestimates (Anderson 2022). Regardless of approach, all rely on accurate geographic range estimates and georeferencing of occurrence localities.

One technique for estimating the potential range is species distribution models (SDMs; sometimes also referred to as ecological niche models), which use occurrence localities and environmental data to predict abiotic suitability across geography (Peterson and Soberón 2012). However, SDMs typically overestimate the species' true distribution, even after considering dispersal limitations. Supplying additional habitat information for post‐processing can refine predictions to estimate the *current* range (Gomes et al. 2019; Merow et al. 2022a). For example, a recent study on a Malagasy rodent improved range estimates by temporally matching occurrence localities and forest cover data to determine a deforestation tolerance threshold (hereby DTT; the lowest tree cover percentage sufficient for the species) and then using it to remove areas of insufficient forest cover at present (Gavrutenko et al. 2021). However, that study did not consider possible georeferencing errors when matching occurrence records to the forest cover data. We use the temporal matching approach as a stepping stone for employing a neighborhood processing method to reflect the uncertainty of geographic coordinates (see below). Our aim is to improve the ecological realism of conservation assessments by ameliorating the effects of georeferencing errors, uncertainty, and mismatched spatial resolutions, to provide more reasonable quantifications of species' actual ranges.

Here, we extend existing post‐processing methodologies to consider the idea of coordinate uncertainty of occurrence localities in relation to the pixel resolution of environmental data. Locality information assigned to occurrence records may not reflect where a species actually exists, but rather an approximation of that locality (e.g., a GPS was not available for older records; or a single set of coordinates was assigned for all records from various sublocalities). Also, advancements in technology have led to finer pixel resolution of remotely sensed data, which may not match other products used with it (e.g., satellite vegetation index products vs. climatic data). Considering this, we propose, apply, and enable a simple approach to resample remotely sensed or other habitat data for post‐processing SDMs, to characterize the area surrounding occurrence records more realistically (i.e., the “neighborhood”). This neighborhood approach is advantageous to balance different spatial resolutions of data being analyzed (e.g., occurrence record uncertainty values, climate data, other abiotic habitat data). This is particularly useful in environments with low spatial autocorrelation, such as mountain ranges, where a small georeferencing error may lead to great differences in habitat information.

**FIGURE 1:**
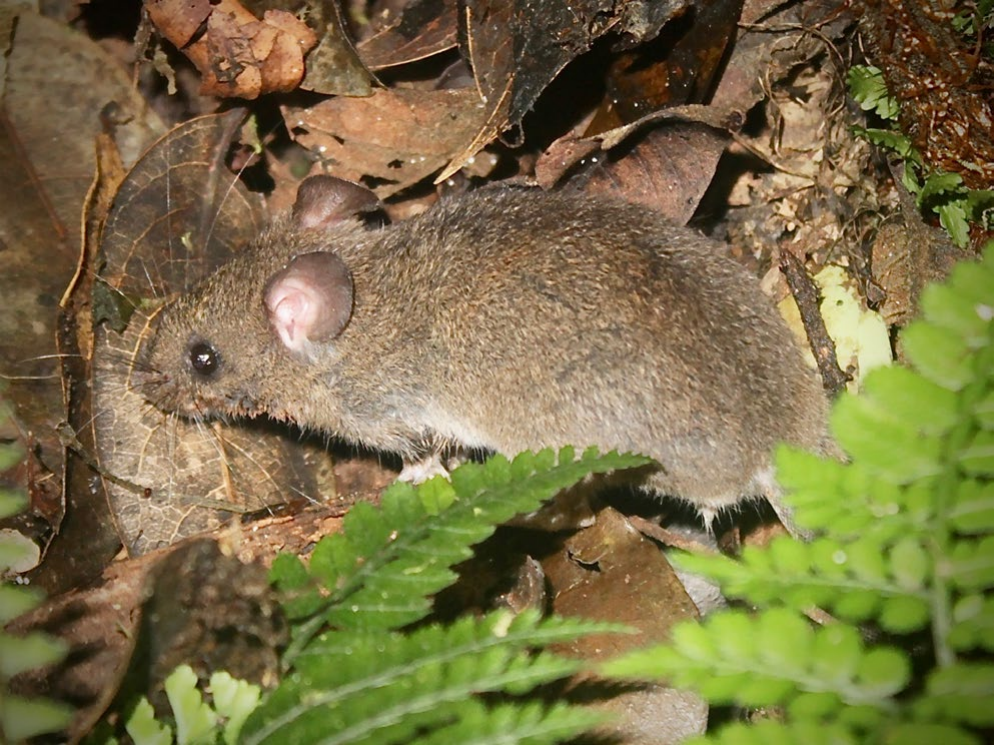
Photograph of *Handleyomys chapmani*, taken by G.E. Pinilla‐Buitrago.

As an example, we use this neighborhood approach in the context of a conservation assessment for *Handleyomys chapmani* (O. Thomas, 1898) a forest‐dwelling rodent endemic to Mexico (Figure 1). It is found in altitudes of 1000–2500 m in the cloud and pine‐oak forests of the Sierra Madre Oriental and Oaxacan Highlands, implying an ecological need for closed‐canopy forest and climatically related altitudinal constraints (Cano and Guevara 2021; Almendra et al. 2018; Ceballos 2013). Basic natural history information known for the species indicates it as a dietary generalist not thought to be strongly affected by particular predators or pathogens (Ceballos 2013). This species' taxonomic understanding had been obfuscated by confusion regarding closely related evolutionary lineages, and recent studies suggest a need for conservation status reevaluation (Almendra et al. 2018, 2014). It is considered threatened under Criterion B of the IUCN (Vulnerable B2ab(ii, iii); Vázquez 2018), based on an AOO below 2000 km^2^, three known locations, and a continuing decline in area and quality of habitat (Vázquez 2018; IUCN 2022). Some of these statements contradict both a prior taxonomic work (Almendra et al. 2014) and later study of the species' range (Cano and Guevara 2021; Almendra et al. 2018), including a larger occurrence dataset.

This species is also opportune for the study because of the availability of recent high resolution forest cover data and its altitudinal associations, which correspond to a highly variable environment within short distances. We first used species distribution modeling to predict a climatically suitable range for the species within a region lacking major dispersal barriers. Using recent occurrence records and forest cover data, we then determined forest cover thresholds (Gavrutenko et al. 2021) from both exact and neighborhood‐processed forest cover data. Next, we used these respective thresholds to remove areas with insufficient forest cover from the predicted suitable range, highlighting areas of inferred deforestation and therefore range loss. We then calculated EOO in two ways; based upon occurrences, and from the two masked suitability maps. From each of these, we also calculated the upper and lower bounds of AOO. From these metrics, we reassessed the threat status for the species. The analyses address the feasibility of post‐processing distribution maps via the proposed neighborhood approach, illustrate them within the context of IUCN Red List assessments, and highlight the importance of spatial grain for conservation uses, in a way highly applicable to other species and scenarios.

## 2 Methods

### 2.1 Species Selection

To demonstrate the feasibility of neighborhood processing of remotely sensed data to refine distribution maps, we selected a montane, forest‐dwelling species with substantial recent records (collected since 2000, amenable for matching with available annual vegetation data). We obtained 116 occurrence records (Appendix S1) of *Handleyomys chapmani* from a recent study of the species' range (Cano and Guevara 2021). We further inspected two of these records because they showed extremely low modeled suitability values based on preliminary species distribution models. The associated georeferences corresponded to sites of the dry interior high plateau uncharacteristic for this species, likely representing either misidentifications or incorrect georeferences. Because they did not match the species' known natural history and environmental associations, we removed them from subsequent analyses (Soley‐Guardia et al. 2014).

### 2.2 Species Distribution Modeling

Species distribution modeling was performed within the software *Wallace EcoMod* (R‐package “wallace”; Kass et al. 2022; Kass et al. 2018; R Core Team 2022), hereafter *Wallace*. To reduce effects of sampling bias, we applied 10 km spatial thinning using the R‐package “spThin” (Aiello‐Lammens et al. 2015) within *Wallace*. Thinning distance followed other studies in this system (Guevara et al. 2018), given the level of spatial sampling bias for small nonvolant mammals in the region, the species' dispersal limitations, and consideration of the elevational gradients and environmental homogeneity of the area. After spatial thinning the 116 records, 41 occurrences remained for final modeling (Appendix S1). For environmental predictors, we used four bioclimatic variables with a 30 arcsecond resolution from the CHELSA v1.0 database (Karger et al. 2018, 2017): maximum temperature of the warmest month (bio05), minimum temperature of the coldest month (bio06), precipitation of the wettest month (bio13), and precipitation of the driest month (bio14). These capture major climatic conditions of the species' range and have been used in previous studies of cloud forest species in the region (Cano and Guevara 2021; Guevara et al. 2018).

**FIGURE 2:**
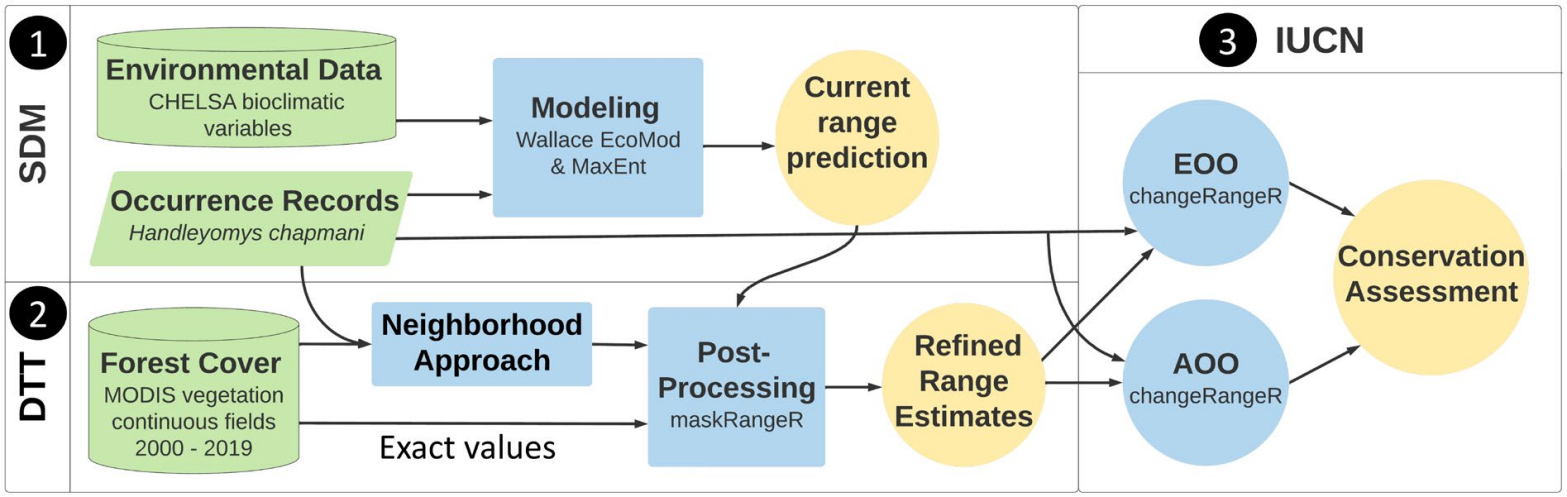
Flowchart of the methodologies employed. Green represents data, blue denotes analyses, and yellow signifies products. Step 1 (**SDM**): A species distribution model (SDM) was built. Step 2 (**DTT**): The output from the SDM was post‐processed two times independently, to remove areas with forest coverage below two respective deforestation tolerance thresholds (DTTs): one using the exact forest cover data, and the second using the neighborhood approach to process forest cover data. Step 3 (**IUCN**): The post‐processed SDMs were then used along with the occurrence data for calculating Extent of Occurrence (EOO) and Area of Occupancy (AOO) for the International Union for the Conservation of Nature (IUCN) assessments.

A species distribution model for *H. chapmani* was built using Maxent (Phillips et al. 2006). We delimited the study region as a 0.5° buffer around localities (to include a variety of environments but avoid including regions without records of the species because of low sampling or barriers to dispersal; Soley‐Guardia et al. 2024) and sampled the full background of 131,680 pixels to provide a comprehensive representation of the environments available within the extent (i.e., avoid missing any rare conditions, which could lead to artifactual environmental truncations of modeled responses; Guevara et al. 2018). Harnessing the functionality of the “ENMeval” 2.0 R‐package (Kass et al. 2021b) and the “maxnet” R‐package (Phillips 2021; Phillips et al. 2017) included in *Wallace*, we delineated training and validation data with a block spatial partitioning method (*k* = 4 folds), and models were built with varying complexity. Specifically, linear; linear‐quadratic; hinge; and linear‐quadratic‐hinge feature class combinations were employed with regularization multipliers from 1 to 5 with a 0.5 step value, generating 36 candidate models. The model performance metrics output by *Wallace* (Appendix S2) include the Akaike Information Criterion corrected for small sample size (AICc), training and validation Area Under the Curve values (AUC.train & AUC.val), and omission rates (OR). We selected the optimal model based on the lowest AICc, while also inspecting the average validation AUC values and omission rates (Guevara et al. 2018; Anderson and Gonzalez 2011), all performance metrics commonly used with Maxent modeling. After selecting the optimal model, we made a cloglog‐transformed continuous prediction and applied the minimum training presence threshold to convert it into a binary (i.e., suitable vs. unsuitable) range prediction (Figure 2, top left panel).

### 2.3 Forest Cover and Neighborhood Processing

**FIGURE 3:**
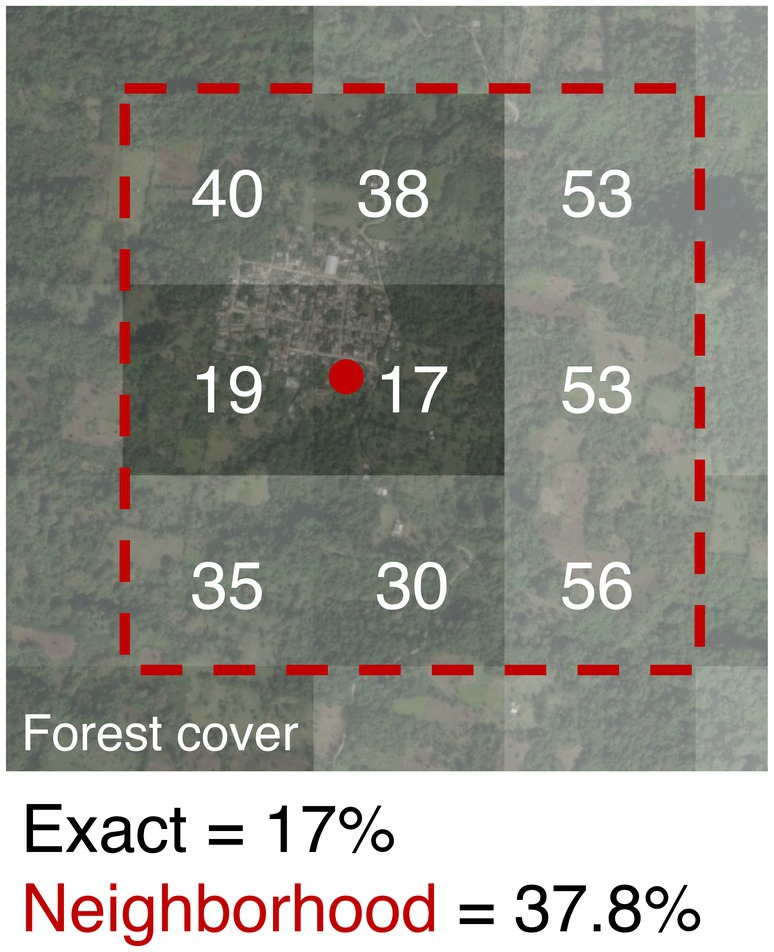
Example of neighborhood approach to processing environmental data. To extract forest cover values for occurrence records (red circle; shown here is record MZFC 11106 in Manuel Gutiérrez Nájera, Veracruz [see Appendix S1]), two approaches were used. The exact method extracted the value of the pixel where the record fell from the raw, unprocessed forest cover layer (values shown in white; here, 17%). In contrast, the neighborhood approach involved processing the forest cover layer so that each pixel was a better representation of the surrounding space but maintained the original data resolution. This study used the mean of a 3 × 3 grid (red dashed line); here, the neighborhood value was 37.8%.

We acquired annual vegetation layers of percent tree cover for each year from 2000 to 2019 (the most recent at the time of analyses). These derived from the NASA Moderate Resolution Imaging Spectroradiometer (MODIS) aboard the Terra satellite. The MOD44Bv006 vegetation continuous fields (VCF) yearly product, which has a 250 m pixel resolution, is generated from monthly composites of 500‐m surface reflectance data (DiMiceli et al. 2015). The compositing removes cloud cover and cloud shadow, an advantage for assessing vegetation in areas of frequent cloud cover. These layers were downloaded from NASA's Land Processes Distributed Active Archive Center (LP DAAC) open‐source data pool as two tiles for Mexico (h8v6 and h8v7) and reprojected to WGS84 and World Cylindrical Equal Area projection. Coordinate uncertainty (e.g., from coordinates reconstructed by prior workers for the records lacking GPS readings, or other GPS reading issues) is sometimes available for occurrence records. While our dataset lacked documented coordinate uncertainty, we considered possible errors in latitude and longitude, including mention of locality descriptions of “by road” distances from populated towns (Appendix S1). From this, also considering the natural history of the species, we determined a 750 m distance from the georeference to be reasonable as a first attempt to illustrate the neighborhood approach for addressing these problems. Accordingly, we resampled each of the yearly forest cover layers individually so that each new pixel value became the mean of itself and the surrounding eight pixels (a 3 × 3 pixel grid, herein referred to as the “neighborhood”), using the “focal()” function from the R‐package “terra” (Hijmans et al. 2023). This maintained the layers at the original 250 × 250 m resolution, but the values of the pixels in this new set of rasters represent the composite of those from a 750 × 750 m neighborhood (Figure 2, bottom left panel; Figure 3).

### 2.4 Determining Deforestation Tolerance Thresholds (DDTs)

For this stage, we subset occurrence records for *H. chapmani* to include only those collected between 2000 and 2019, to correspond to the forest cover layers available at the time of analyses. This included localities from the initial dataset, as well as additional records not used in modeling due to: identical coordinates but with different collection dates; different coordinates falling within the same climatic data cell as another record; or removed by the spatial thinning (Appendix S1). Note that although we conducted spatial thinning for the occurrence dataset used in modeling the species' distribution, the DTTs and the associated neighborhood approach should not be strongly affected by sampling bias. Because of this, and to retain the maximum number of datapoints possible, for this part of the analysis records were not thinned spatially.

To extract the values of forest cover for the place and time where each record fell, we temporally matched the year of collection with the corresponding annual forest cover layer (separately for the exact unprocessed layers and the neighborhood‐processed ones; Figure 2, bottom left panel). Since *H. chapmani* is a forest‐dwelling species, localities with an extremely low forest cover percentage may indicate problematic coordinates or issues within the forest cover data itself. Therefore, after sorting by forest cover percentages, we visually inspected the localities associated with the lowest ones using Google Earth imaging (https://earth.google.com) and Esri World Imagery (Esri 2023) to check for inconsistencies. For each dataset (exact and neighborhood), we then eliminated any localities with aberrantly low forest cover values (Figure 2, bottom left panel).

### 2.5 SDM Post‐processing & Range Calculations

Using the DDTs derived from each of the two methods, along with the respective forest cover layer for 2019 (the most recent one considered), we post‐processed the range prediction to include only areas both climatically suitable and with sufficient forest cover. This was done by masking using the R‐package “maskRangeR” (Merow et al. 2022b). From each of these resulting maps, we calculated EOO and the upper and lower bounds for AOO with the R‐package ‘changeRangeR’ (Galante et al. 2022), QGIS v3.1.2 (QGIS.org 2023), and/or calculations with the “terra” R‐package (Hijmans et al. 2023). EOO was measured in two ways: first from a minimum convex polygon drawn around the occurrence records; and second from a minimum convex polygon around the binary suitability prediction (each conducted before and after the two respective masking procedures).

For AOO comparison, we calculated analogous areas of suitability at the finer spatial resolution (which matches the forest cover data), as well as after the suitability map and its two masked versions were reprojected into a World Cylindrical Equal Area projection with a coarser resolution of a 2 × 2 km grid, which is standard for AOO calculations (IUCN 2022). Lower bound AOOs were calculated by totaling the grid cells corresponding to occurrence records, both before considering sufficient forest cover and then again after having removed records below the respective DTT threshold of each habitat‐masked suitability prediction. Upper bound AOO estimates were derived from the sum of all cells in the grid corresponding to the respective range predictions, before and after post‐processing by each masking method (Figure 2, right panel).

## 3 Results

### 3.1 Species Distribution Modeling

**FIGURE 4:**
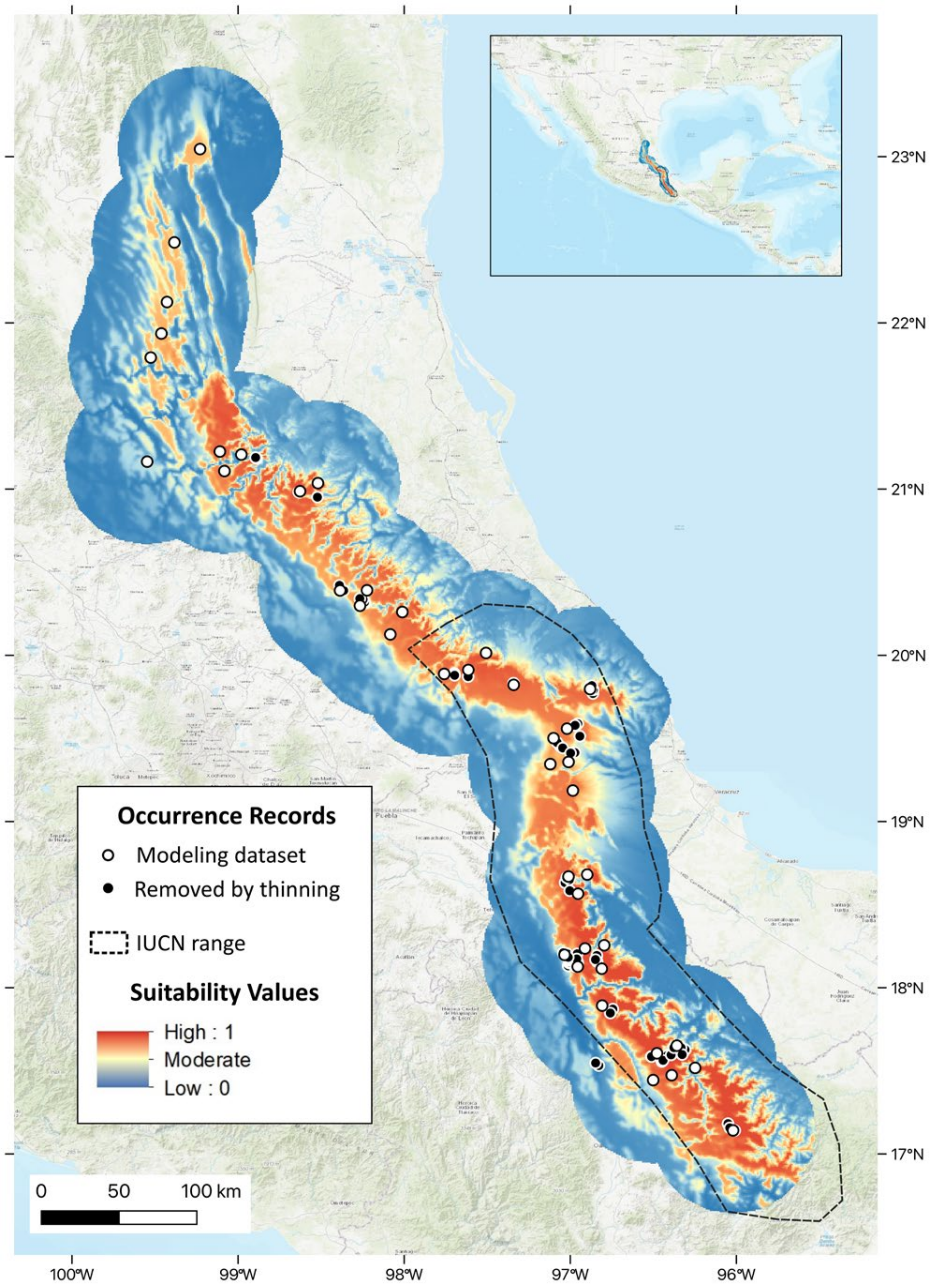
Continuous suitability prediction from species distribution modeling for *Handleyomys chapmani*. Red indicates the highest suitability, while blue denotes the lowest. Occurrence localities are shown as circles; those in white indicate the data used for modeling after 10 km thinning; black circles indicate additional localities removed via thinning. The current range reported by the International Union for the Conservation of Nature (IUCN; Vázquez 2018) is represented by the dashed polygon. Basemap is ESRI World Topo EPSG:4326 (Esri 2023).

We obtained a high performing and ecologically realistic species distribution model for *Handleyomys chapmani*. Based on the lowest Akaike Information Criterion corrected for small sample size (909.77), we chose the Hinge feature class with a regularization multiplier of two from the resulting candidate models (Appendix S2). The model for these settings corresponded to the second highest average validation AUC value of the candidate models (only 0.0041 lower than the highest), and a low average validation omission rate at the minimum training presence threshold (0.023 ). In addition to quantitatively good performance, it also output an ecologically realistic prediction concordant with similar studies of the species (Cano and Guevara 2021). The continuous prediction showed high suitability along the Sierra Madre Oriental into the northern Sierra Madre del Sur, with values generally decreasing downslope (Figure 4). Applying the minimum training presence threshold (values < 0.23 as unsuitable) emphasized the striations in the complex terrain of the northernmost part of the range, and there were other indications of natural fragmentation throughout the distribution (e.g., lower and/or drier passes).

### 3.2 Determining Deforestation Tolerance Thresholds (DDTs)

**FIGURE 5:**
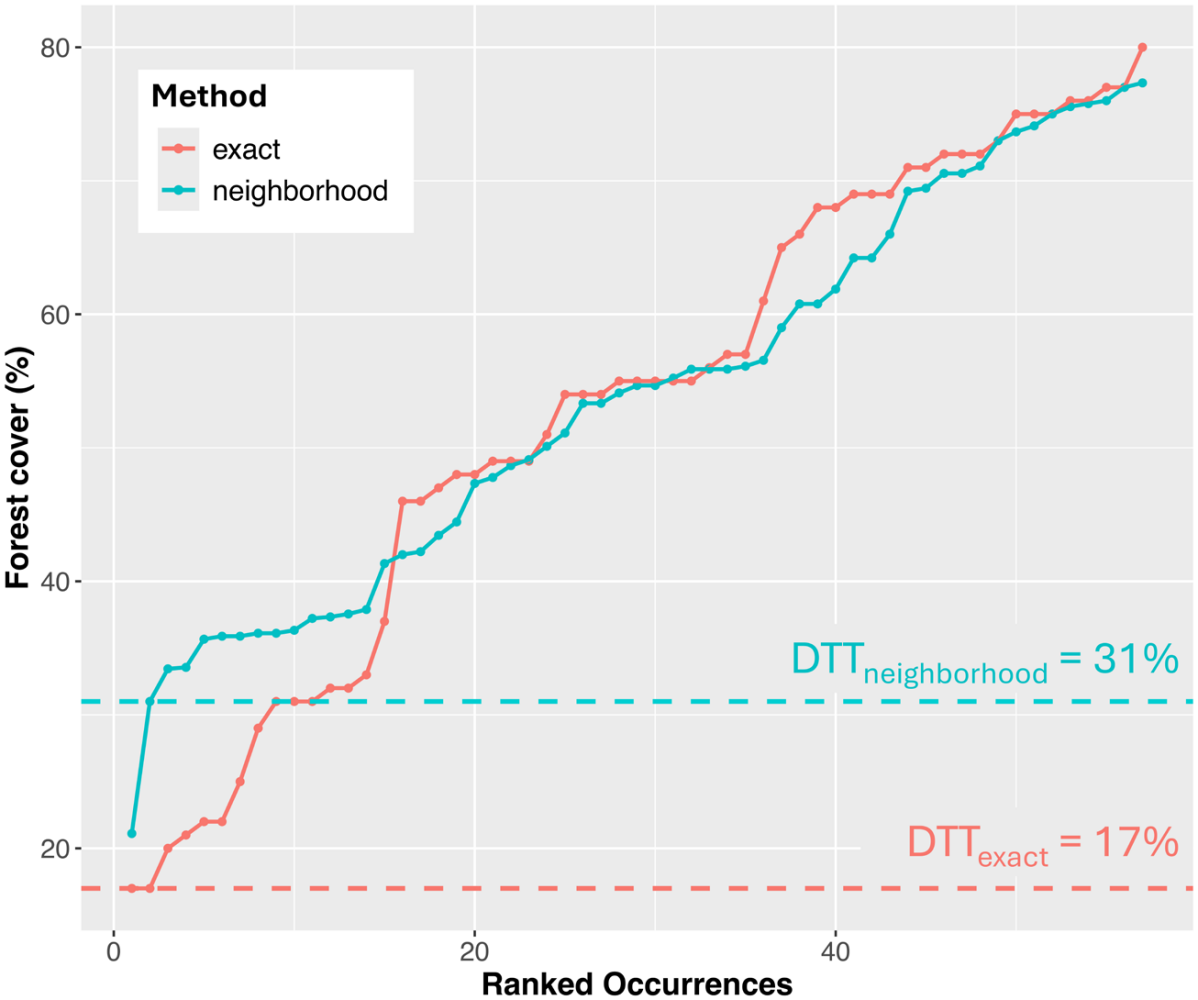
Forest cover values corresponding to temporally matched occurrence records of *Handleyomys chapmani* for the two methods (exact and neighborhood‐processed) to determine deforestation tolerance thresholds (DTTs). On the x‐axis, occurrences are ranked by method for comparison (note, the locality with the lowest value from the exact method does not necessarily correspond to the same one for the neighborhood). These thresholds, 17% and 31%, were then used in post‐processing to remove regions that fall below the thresholds.

In total, 57 recent records collected between 2000 and 2019 were used to determine DDTs (Appendix S1). The lowest attributed forest cover percentages using the exact data were two records tied at 17% (Figure 5, orange). One was collected in 2010 from the town of “Manuel Gutiérrez Nájera” and the other in 2013 from “28.5 NE of Tetela de Ocampo (by road), Puebla”. For the neighborhood data, the lowest was 21.1% collected in 2001 from “6 km SW de Xicotepec of Juárez (by road), Puebla” followed by a jump to 31% for the second lowest, collected in 2006 near “El Durazno Ixtlahuaca, Tezuitlán, Puebla” (Figure 5, blue). Upon inspection using Google Earth imaging and Esri World Imagery, we determined that information for two of the lowest scoring localities, one for each approach, did not match general knowledge of the species' known natural history. Rather, we inferred that the true sites of sampling likely correspond either to extremely small patches of remnant vegetation (under the 0.25 km pixel size of the forest data) or to a place in the general vicinity of the assigned coordinates (i.e., only very approximate match with the coordinates currently assigned to the record). For the exact approach, the lowest locality (17%; Figure 5) fell into a pixel that included the edge of a town. The second lowest, also 17%, fell in a pixel including a farm and associated buildings interspersed with intact forest. For the neighborhood approach, the record with the lowest forest cover value (21.1%; Figure 5) corresponded to a pixel that was diagonally transected by a major highway, but the second lowest (31%; Figure 5) represented a forest expanse surrounding a nearby town. The record with the lowest value for exact forest cover was not the same one as the lowest for the neighborhood approach, a notable difference across scales. For each approach, we interpret a record with the lowest value as uncharacteristic of the species' natural history. In contrast, the second lowest value for each approach comprised a mosaic of forested and nonforested areas, plausible for the species. Therefore, we dropped the lowest value for each approach and chose the second lowest one as the respective DTT instead (17% exact, 31% neighborhood; Figure 5).

### 3.3 SDM post‐processing and Range Calculations

**FIGURE 6:**
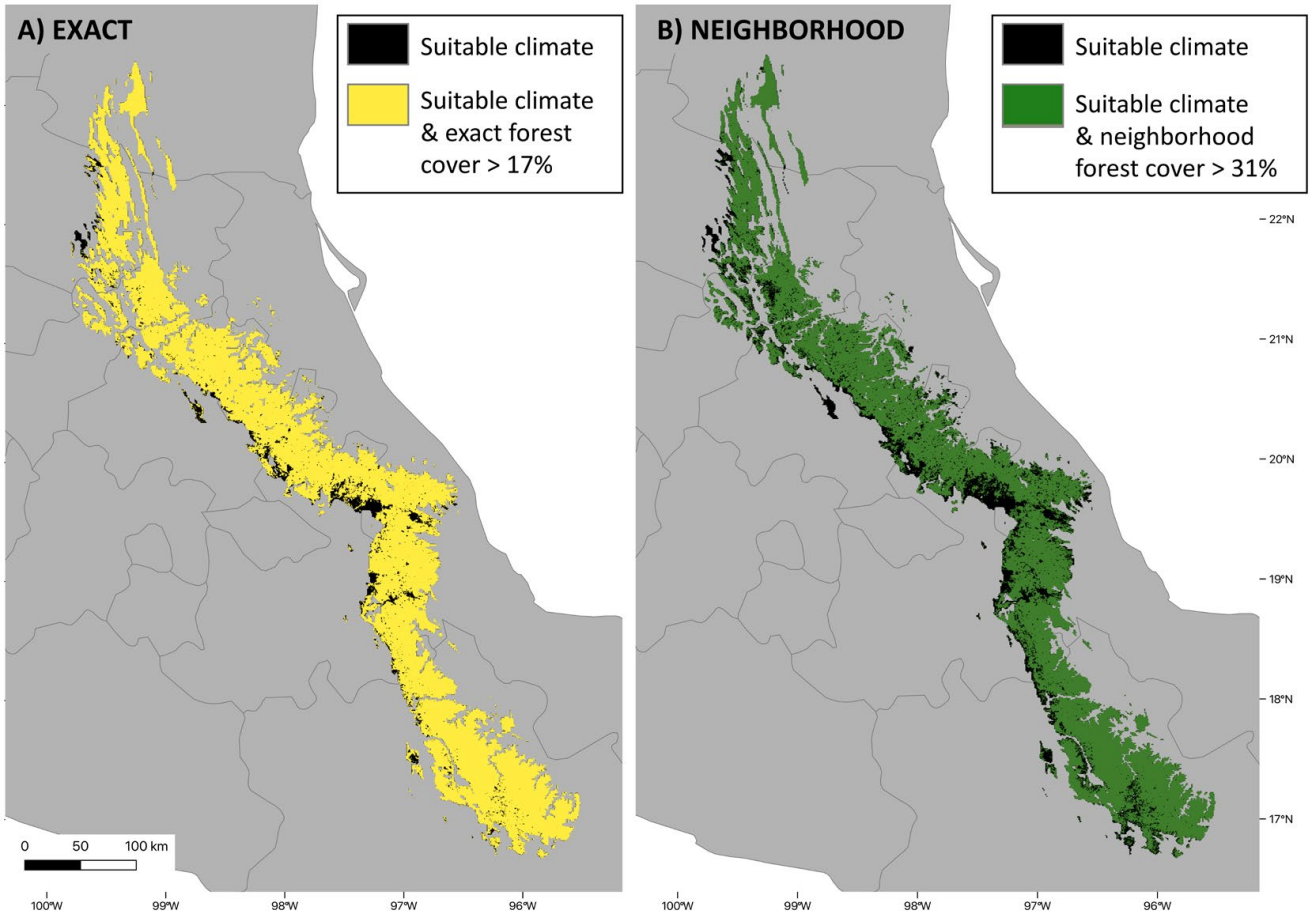
Post‐processed climatic suitability maps for *Handleyomys chapmani*. Areas removed were those that fell below respective deforestation thresholds for 2019 determined in two ways: using the exact value as a threshold and the raw forest cover data (A; yellow), and using forest cover processed with the proposed neighborhood approach and subsequent threshold value (B; dark green). Note greater proportional loss for the neighborhood approach.

**FIGURE 7:**
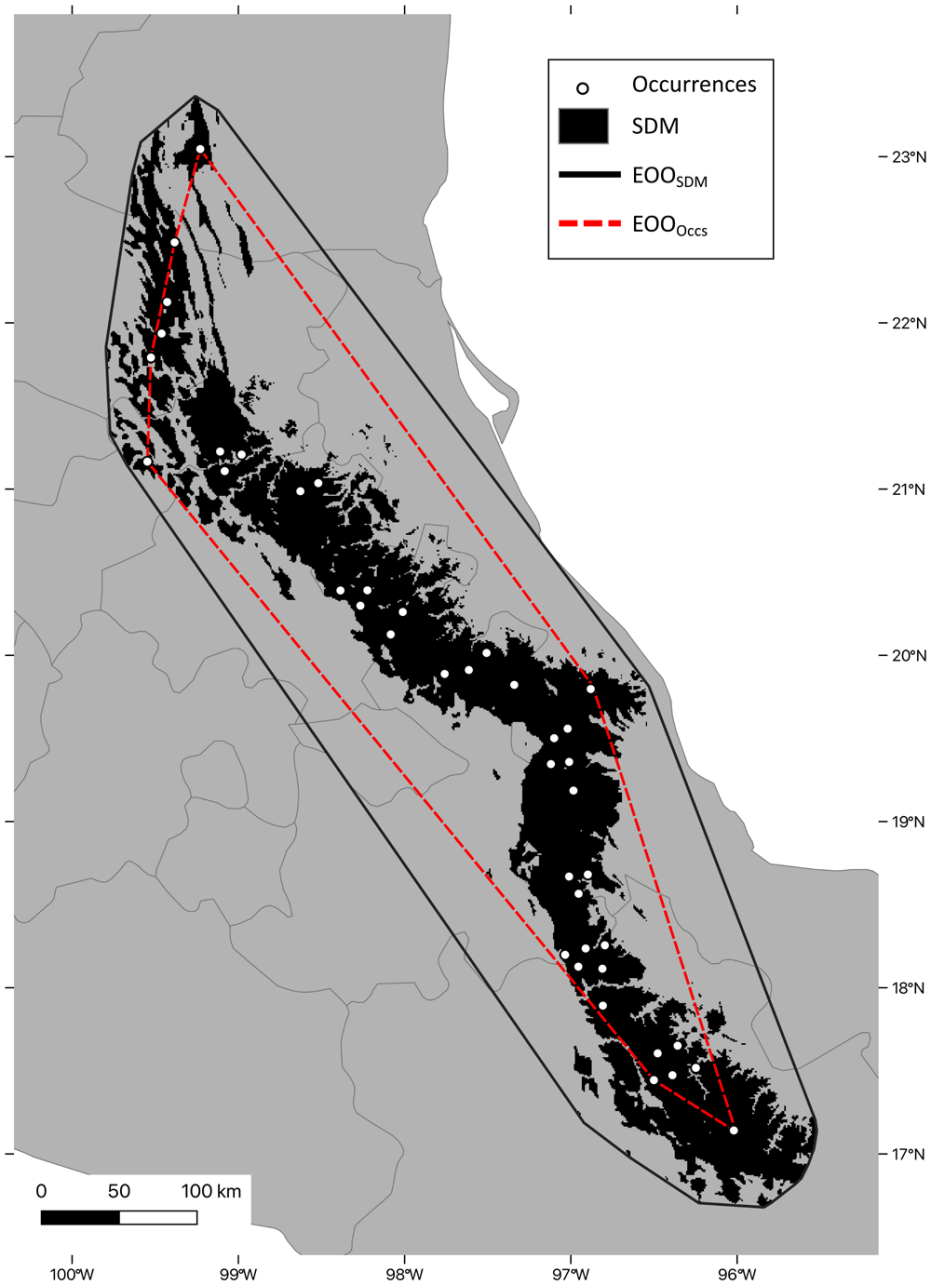
Comparison of Extent of Occurrence (EOO) for *Handleyomys chapmani*, calculated from a minimum convex polygon surrounding occurrence localities (red), and around suitable habitat predictions within the range (black).

The climatically suitable range of *H. chapmani* was reduced when using both the DTT of 17% with the exact forest cover data (Figure 6A), and the DTT of 31% with the neighborhood‐processed forest cover data (Figure 6B). While there was a drastic disparity in the area of the EOOs calculated from occurrence records versus from the modeled range (Figure 7), much less difference existed between the exact and neighborhood methods of masking (Table 1). Despite the removal of some older records falling into areas of current low forest cover, the estimates for EOO based on localities were the same for both masking methods, as well as without any post‐processing (72,513 km^2^). Additionally, when calculating the EOO from a polygon around the modeled range estimates, the maps before and after post‐processing with the exact forest cover were similar (129,029 and 129,015 km^2^, respectively) but reduced by approximately 1000 km^2^ when using the neighborhood approach (128,067 km^2^).

**TABLE 1:** Table of conservation metrics values.

Post‐processing method	Occurrence records	Area of range (km^2^)	Extent of Occurrence		Area of Occupancy	
			Localities (km^2^)	Habitat map (km^2^)	Occupied cells (km^2^)	Suitable habitat (km^2^)
Full dataset/SDM (no post‐processing)	114	42,894	72,513	129,029	388	54,016
Exact DTT = 17% (SDM – raw forest cover < 17%)	112	40,341	72,513	129,015	380	53,240
Neighborhood DTT = 31% (SDM – neighborhood‐processed forest cover < 31%)	108	36,948	72,513	128,067	368	50,580

*Note:* The full occurrence dataset (corresponding to the 114 occurrence records for *Handleyomys chapmani*) and the species distribution model prediction (SDM) were post‐processed two ways: (1) The exact method used 17% as a deforestation tolerance threshold (DTT); any occurrences or pixels falling into areas with lower forest cover for 2019 were removed. (2) The neighborhood approach used the neighborhood‐processed forest cover layer for 2019 to remove any records or pixels falling below the neighborhood DTT of 31%. The Area of Range (km^2^) was calculated at the resolution of the forest cover data (0.25 × 0.25 km) in World Cylindrical Equal Area projected coordinate system. Minimum convex polygons were drawn around the occurrence localities, or the respective suitability map, to calculate estimates of Extent of Occurrence. Lower bounds of Area of Occupancy were calculated by summing the occupied cells of a 2 × 2 km grid corresponding to the respective records. The upper bounds of Area of Occupancy were calculated from the 2 × 2 km grid cells with both suitable climate and habitat (separately for each post‐processing method); this assumes that the species occupies all suitable areas with habitat remaining.

Estimates of AOO varied greatly between lower and upper bounds but were rather similar among post‐processing approaches. The lower bound of AOO, estimated from cells documented as occupied by occurrences currently holding sufficient forest via the respective post‐processing methods, was only 368–380 km^2^ (and slightly higher at 388 km^2^ with no consideration of current forest cover; Table 1). The upper bound of AOO, estimated from cells holding suitable climate and sufficient forest cover, was 53,240 km^2^ when using the exact method and 52,580 km^2^ using the neighborhood approach (and somewhat higher without habitat masking; Table 1).

## 4 Discussion

### 4.1 Threat Level Reassessment

Estimates of EOO and the most reasonable of those for AOO suggest removing *Handleyomys chapmani* from any threatened category. Regardless of approach, the EOO quantifications are far larger than the previous characterization of 49,406 km^2^ (Vázquez 2018), due to the inclusion here of many additional occurrence localities in the northern part of the range. All put the species outside the Vulnerable category based on EOO for Criterion B since they are above 20,000 km^2^ (IUCN 2022). Lower and upper bound estimations of AOO from this study lead to a span from a dire scenario to delisting the species from being threatened. The lower bound AOO values would qualify the species as Endangered under Criterion B2 (< 500 km^2^, if necessary subcriteria are met; IUCN 2022), but they presumably are strongly biased underestimates due to sparse sampling throughout the region. In contrast, the upper bound measurements of AOO (under either the exact or neighborhood approach) put the species outside of any threatened category, even Vulnerable (< 2000 km^2^; IUCN 2022), assuming the species inhabits all suitable areas still holding habitat within its range. Even highly likely values of prevalence (i.e., proportion of sites currently containing habitat that a species occupies within its range; Anderson 2022), for example 0.5 and above, would lead to an estimated AOO far above the 2000 km^2^ threshold.

Based on the conservation metrics reported here, we suggest the current placement of the species in the vulnerable category is inappropriate and misdirects conservation focus, since the estimates of both EOO and AOO generated here put the species outside of any threatened category. However, despite suggesting the delisting of the species, this study does pinpoint locations where deforestation has reduced its range, as well as areas of both climatic suitability and sufficient forest worth protecting for conservation planning. Such spatial details may be useful at a regional extent, such as for managing individual national parks or other protected areas.

**FIGURE 8:**
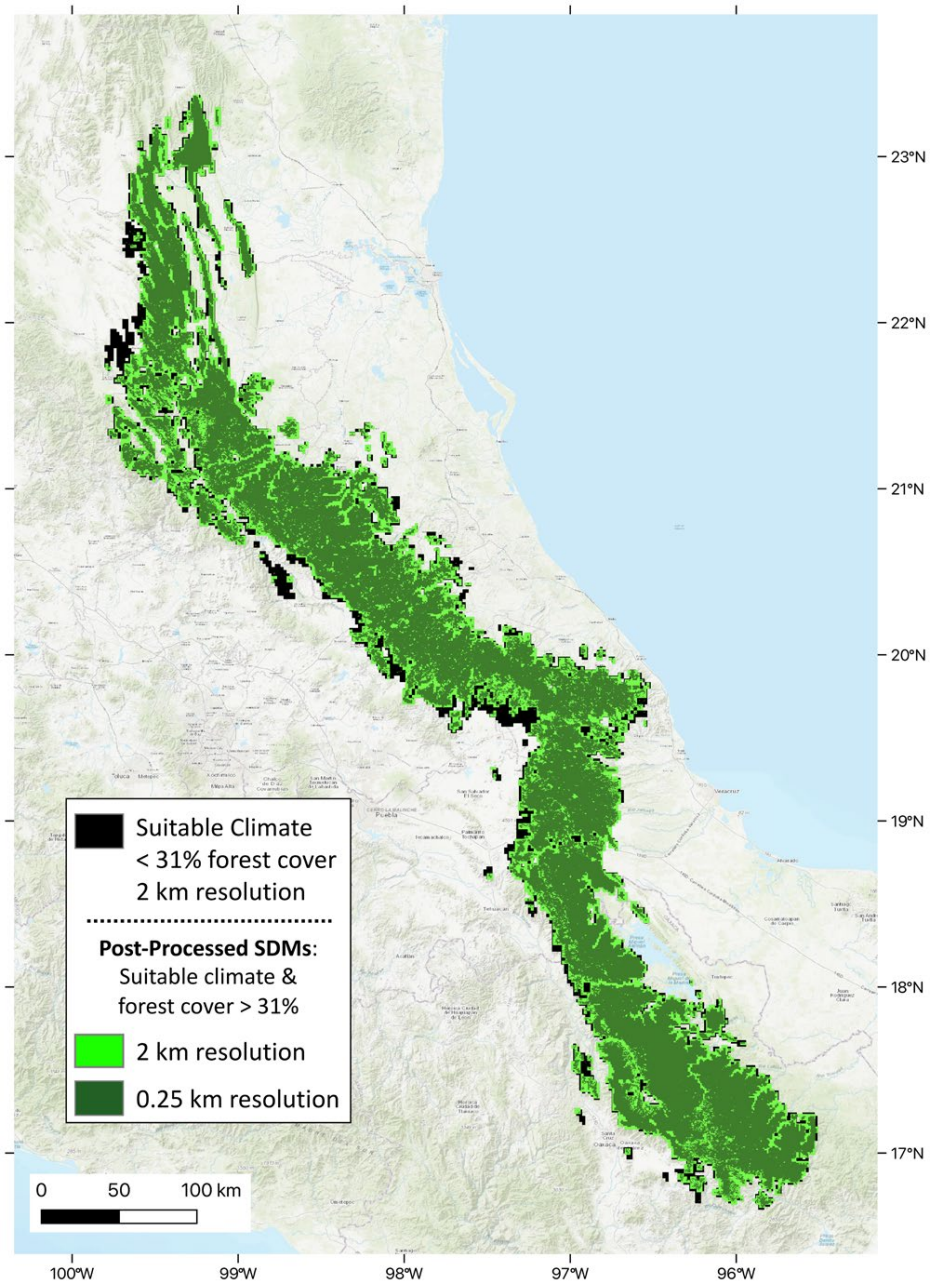
Post‐ processed species distribution model predictions for *Handleyomys chapmani*. Post‐processing and resampling led to substantial differences in spatial patterns and areal estimates at different resolutions (example shown here via the neighborhood method using a deforestation tolerance threshold of 31%). Black indicates climatically suitable areas under the deforestation tolerance. Green indicates parts of the suitability prediction that held sufficient forest cover, with dark green at the native resolution of the forest cover data (0.25 × 0.25 km), and light green resampled to a 2 × 2 km resolution. Note that inferred range loss is much less for the coarser resolution (black) than for the finer size (black plus light green), with completely different spatial patterns of inferred loss. Basemap is ESRI World Topo EPSG:4326 (Esri 2023).

Additionally, we found a noteworthy distinction in the spatial and proportional patterns of loss at the different spatial resolutions. While the areas with insufficient forest removed from the climatic suitability prediction were generally similar throughout the extent of the species' distribution, the most notable inferred loss occurred on the western slopes of the range, especially between northern Puebla and Veracruz states (Figure 6, black). At the grain of the forest cover data (0.25 km), the inferred loss of climatically suitable area from the binary SDM prediction was 2553 km^2^ using the exact method and 5946 km^2^ for the neighborhood approach (Table 1). In contrast, the estimated range lost when resampled to the IUCN standard 2 × 2 km resolution was only 776 and 3436 km^2^, respectively. Importantly, by converting to the 2 × 2 km grid, many of the spatial patterns of current vegetation are erased and some of the insufficiently forested areas detected within the range are not visible at the coarser resolution (Figure 8). For example, in the neighborhood masked map, much of the inferred range loss (Figure 8, dark green) at the finer grain is undetectable when transformed to the coarser resolution (Figure 8, bright green). This discrepancy across resolutions is prevalent throughout the range, particularly along the valleys, where finer resolution data pick up lower levels of forest cover.

### 4.2 Utility of Neighborhood Approach

More generally, the neighborhood approach for post‐processing suitability predictions is a transparent way to include relevant habitat data as an additional post‐processing step in species distribution modeling and may be helpful when models are used for conservation assessments (Merow et al. 2022b). Particularly when used to mask a distributional estimate from an SDM (as here), it is more replicable than using subjective expert‐drawn maps (Merow et al. 2022a). More generally, it enhances existing pathways for considering recent habitat information and biotic interactors in post‐processing, especially for relatively data poor species (Galante et al. 2022; Gavrutenko et al. 2021; Gomes et al. 2019; Kass et al. 2021a; Merow et al. 2022a).

Nevertheless, specific implementations of the neighborhood processing and associated methodologies explored here of course hold various limitations. Critically, they depend on either data or assumptions regarding coordinate uncertainty (e.g., here the 750 m neighborhood chosen). Fortunately, current data collection protocols tend to include such information, for example via community science and camera trapping initiatives (Kays et al. 2020), and estimation of coordinate uncertainty via retrospective georeferencing (e.g., of older museum and herbarium specimens) represents a key direction in biodiversity informatics (Anderson et al. 2020; Stein and Wieczorek 2004). Another limitation is access to relevant environmental data sources for post‐processing, as not all are publicly accessible, free, and/or available in compatible formats. However, use of remotely sensed products in distribution modeling (as predictor variables or in post‐processing) has risen substantially and should continue (He et al. 2015). This is helpful since studies using SDMs for conservation are often limited to basing “current” predictions on 30‐year climatic averages, whereas many remotely sensed habitat data are updated annually (DiMiceli et al. 2015). The neighborhood approach can be applied to many terrestrial as well as aquatic species, as well as be used with various relevant datasets beyond forest cover, such as NDVI, soil moisture and acidity indices, ocean temperature, depth, and salinity. We emphasize that the species' natural history (to the degree known) should be taken into account, including consideration of strong biotic interactions. Additionally, because an SDM itself remains subject to many methodological assumptions and decisions, researchers should closely follow best practices and community standards for building, evaluating, estimating uncertainty, and reporting (Araújo et al. 2019; Zurell et al. 2020; Soley‐Guardia et al. 2024).

In this study, we considered forest cover percentages for a forest‐dwelling species to refine the geographic range estimates via EOO and AOO metrics. While some occurrence localities can be considered no longer viable due to too low forest cover, this had no impact on EOO calculations derived from occurrence records (Figure 7; Table 1), largely because the dropped localities corresponded to central areas of the range, rather than the outer edges where a minimum convex polygon's shape would be affected. However, this was not the case for the EOO estimates derived from post‐processing the range map made from the suitability estimate, especially for the neighborhood approach. This reiterates how forest loss along the outer extents of the range can affect the EOO, but any habitat loss within the central range is not captured in these measurements.

Similarly, spatial patterns of deforestation are not fully reflected in calculations of AOO due to its spatial scale. For the lower bound estimates of AOO, masking only led to minor reductions. Even for the upper bounds of AOO, the 2 × 2 km coarsening required by the IUCN removes many intricacies of the inferred loss from deforestation (Figure 8), thereby removing relevant habitat information from such assessments. The AOO value might also be changed depending on where the grid lines are drawn, which would affect estimates for all species, but especially those with low dispersal rates or sensitive to microclimate variation.

The differences in threat level for *H. chapmani* determined by calculating AOO from documented occurrence records versus a suitable habitat approach highlight the importance of selecting appropriate methodologies for calculating AOO, depending on the spatial density of sampling and variation in data resolutions. As remotely sensed data improve and their resolutions get finer, so does the need for higher quality of georeferencing, pointing toward the value of continued biological survey efforts. While the occurrence records for this species did not include uncertainty data, databases such as the Global Biodiversity Information Facility (gbif.org) increasingly need to include this information (Anderson et al. 2020). This study emphasizes the importance of accurate georeferencing in the field. Nevertheless, using the neighborhood approach can alleviate some complications from uncertainty, leading to better species range predictions and derived areal estimates, particularly those used for conservation assessments.

### 4.3 Outlook

The neighborhood processing code (Appendix S3) was written in the R‐language and is compatible with relevant packages like “maskRangeR” and “changeRangeR” (Merow et al. 2022b; Galante et al. 2022); hence, it has promise to improve range estimates and conservation assessments when used in conjunction with them. It can also be customized to match resolutions of climatic data or remotely sensed data for the modeling stage itself or for post‐processing analyses, as the size of the “neighborhood” can be changed easily. Here, we used the mean of the 9 pixels, but employing the maximum or minimum value is also possible, which may be useful for calculating proximity to streams or roosting sites, or distance from roads. Varying the neighborhood size and mathematical operation can be adjusted for improved characterization of spatial patterns, particularly in areas with disparate habitat information in close proximity (e.g., mountains, human impacted regions, urban areas). While we focused on a montane region, this can be useful for any area with low spatial autocorrelation or a mosaic of habitats. In conclusion, the general applicability of the neighborhood approach and its simplicity make it a useful tool to make range assessments more accurate and feasible to update over time.

## Author Contributions

**Bethany A. Johnson:** conceptualization (equal), data curation (lead), formal analysis (lead), methodology (equal), visualization (equal), writing – original draft (lead), writing – review and editing (lead). **Gonzalo E. Pinilla‐Buitrago:** conceptualization (equal), formal analysis (supporting), methodology (equal), writing – review and editing (equal). **Robert P. Anderson:** conceptualization (equal), methodology (equal), supervision (lead), writing – review and editing (equal).

## Conflicts of Interest

The authors declare no conflicts of interest.

## Supporting information


**Appendix S1.** Occurrence datasets for *Handleyomys chapmani*.

**Appendix S2.** Table of model performance metrics.

**Appendix S3.** R scripts.

## Data Availability

Data and accompanying R‐code are publicly available on Dryad: https://doi.org/10.5061/dryad.sxksn03ft.
