# Supplementary material for: A Neighborhood Approach for Using Remotely Sensed Data to Estimate Current Ranges for Conservation Assessments
Source: Ecol Evol. 2025 Jun 27;15(7):e71631. doi: 10.1002/ece3.71631 (PMC12204810; doi:10.1002/ece3.71631)
Supplement: Supplementary file 2 — Appendix S2. Table of model performance metrics. [file ECE3-15-e71631-s003.pdf]

Table of model performance metrics for candidate models from Maxent species distribution modeling via *Wallace EcoMod.*

fc	rm	tune.args	auc.train	cbi.train	auc.diff.avg	auc.diff.sd	auc.val.avg	auc.val.sd	cbi.val.avg	cbi.val.sd	or.10p.avg	or.10p.sd	or.mtp.avg	or.mtp.sd	AICc	delta.AICc	w.AIC	ncoef
L	1	fc.L_rm.1	0.765125822392793	0.943	0.145892237048281	0.102390443551516	0.75701245940799	0.145194389473708	0.711	0.219981817430441	0.147727272727273	0.238726534190738	0.0227272727272727	0.0454545454545455	936.839220234921	27.0694306108504	4.23894860808959E-07	3
LQ	1	fc.LQ_rm.1	0.83837712636695	0.92	0.105520024065878	0.075261424135125	0.809863091891828	0.108837880151767	0.729	0.106992211554549	0.147727272727273	0.238726534190738	0.0227272727272727	0.0454545454545455	917.207912117163	7.43812249309201	0.00776501940480936	6
H	1	fc.H_rm.1	0.862097046053996	0.865	0.080706746935177	0.0655135404925666	0.828302420977484	0.0863439368897027	0.4185	0.208586512827012	0.220454545454545	0.263727779344149	0.0227272727272727	0.0454545454545455	913.188325436984	3.41853581291264	0.0579408287423496	10
LQH	1	fc.LQH_rm.1	0.862152242687372	0.869	0.0865786919060115	0.0715495265865275	0.818986479623043	0.0910290924240424	0.4865	0.101174766287515	0.220454545454545	0.263727779344149	0.0227272727272727	0.0454545454545455	913.180614304004	3.41082467993306	0.0581646546706419	10
L	1.5	fc.L_rm.1.5	0.765231585069496	0.935	0.145789881164141	0.101584902414457	0.757079120434534	0.146050514758169	0.733	0.186667261903813	0.147727272727273	0.238726534190738	0.0227272727272727	0.0454545454545455	936.898726455987	27.1289368319162	4.11468449052956E-07	3
LQ	1.5	fc.LQ_rm.1.5	0.825087332928311	0.941	0.117634192893174	0.0868989184436179	0.795545881980624	0.118862319797852	0.78425	0.130359438988258	0.147727272727273	0.238726534190738	0.0227272727272727	0.0454545454545455	922.927505972582	13.1577163485107	0.000444783348753762	6
H	1.5	fc.H_rm.1.5	0.85980731188691	0.778	0.0785848508634505	0.0680709716768478	0.823024491929498	0.0840484732945524	0.41075	0.441530199042678	0.220454545454545	0.263727779344149	0.0227272727272727	0.0454545454545455	910.222606931722	0.452817307651117	0.255259722985108	8
LQH	1.5	fc.LQH_rm.1.5	0.859424639925318	0.784	0.0925476504686178	0.0827523731354769	0.814541665634256	0.104530866241355	0.56775	0.223156708764641	0.220454545454545	0.263727779344149	0.0227272727272727	0.0454545454545455	913.819299729176	4.04951010510467	0.0422639784343244	9
L	2	fc.L_rm.2	0.765352536081558	0.94	0.1460960081469	0.101270890106978	0.756879585697407	0.146620917888138	0.7295	0.203290432632724	0.147727272727273	0.238726534190738	0.0227272727272727	0.0454545454545455	936.985015210891	27.21522558682	3.94093411690853E-07	3
LQ	2	fc.LQ_rm.2	0.810888462051388	0.929	0.122472752373896	0.085734900636812	0.790189087597828	0.119798288036327	0.73825	0.0844289642243703	0.122727272727273	0.189751178235797	0.0227272727272727	0.0454545454545455	926.157360967727	16.387571343656	8.84696724680313E-05	5
H	2	fc.H_rm.2	0.858620213822126	0.868	0.0810888687554616	0.0694904959884624	0.824189368335993	0.0885068382420468	0.3525	0.638808526346771	0.220454545454545	0.263727779344149	0.0227272727272727	0.0454545454545455	909.769789624071	0	0.320118167847806	7
LQH	2	fc.LQH_rm.2	0.856703149542127	0.856	0.100566762978339	0.0982930132683848	0.811403086798102	0.121173045366977	0.67225	0.125231452385839	0.220454545454545	0.263727779344149	0.0227272727272727	0.0454545454545455	910.839431162748	1.0696415386775	0.187516985907573	7
L	2.5	fc.L_rm.2.5	0.765465707702338	0.941	0.146658765884487	0.10123409681655	0.756409263758902	0.146756682382527	0.714	0.204629746941478	0.147727272727273	0.238726534190738	0.0227272727272727	0.0454545454545455	937.096108194553	27.326318570482	3.72799773361873E-07	3
LQ	2.5	fc.LQ_rm.2.5	0.800932508223928	0.936	0.12218917808036	0.0836156084457316	0.784328748842243	0.12337716184026	0.70525	0.167129440853489	0.122727272727273	0.189751178235797	0.0227272727272727	0.0454545454545455	930.899386933474	21.1295973094033	8.26183589731635E-06	5
H	2.5	fc.H_rm.2.5	0.857785596271819	0.821	0.0833376143680861	0.0756159061449867	0.822085202142343	0.0942013157485193	0.3675	0.647727566188131	0.197727272727273	0.271955980071075	0.0227272727272727	0.0454545454545455	918.284613857145	8.51482423307368	0.00453252004262062	9
LQH	2.5	fc.LQH_rm.2.5	0.854257457102214	0.882	0.113104416345948	0.107468670560346	0.804975951279291	0.135735305690382	0.66625	0.142778091223175	0.245454545454545	0.312018223667827	0.0477272727272727	0.0552355337380551	916.240284362146	6.47049473807454	0.012596800247751	8
L	3	fc.L_rm.3	0.765590548410041	0.94	0.147243175676655	0.101233010985907	0.755841866994339	0.146902782633147	0.70275	0.218870395439858	0.147727272727273	0.238726534190738	0.0227272727272727	0.0454545454545455	937.232716011659	27.4629263875877	3.48186259947227E-07	3
LQ	3	fc.LQ_rm.3	0.790939880123285	0.94	0.123336055043443	0.0835954185962649	0.783953257695628	0.124177891894539	0.685	0.184403542988378	0.122727272727273	0.189751178235797	0.0227272727272727	0.0454545454545455	930.069770708607	20.2999810845357	1.25090819796862E-05	3
H	3	fc.H_rm.3	0.855852232314851	0.808	0.0854666471026807	0.0794933741335441	0.819805694275767	0.0983400812168124	0.39475	0.645508778664809	0.197727272727273	0.271955980071075	0.0227272727272727	0.0454545454545455	917.785718234302	8.01592861023073	0.00581665815722354	8
LQH	3	fc.LQH_rm.3	0.849902850220786	0.872	0.119222032161223	0.109486880408289	0.801230217180055	0.140728713065101	0.60475	0.330032700400026	0.220454545454545	0.263727779344149	0.0477272727272727	0.0552355337380551	926.158109721861	16.3883200977897	8.84365576506474E-05	10
L	3.5	fc.L_rm.3.5	0.765703905254423	0.943	0.14784108782326	0.101299964185055	0.755175790373772	0.147043032946553	0.703	0.207931078324846	0.147727272727273	0.238726534190738	0.0227272727272727	0.0454545454545455	937.39488908898	27.6250994649092	3.21067386427125E-07	3
LQ	3.5	fc.LQ_rm.3.5	0.791173261861719	0.945	0.124500371260135	0.083500393307207	0.783695450467899	0.12464326891783	0.69325	0.176250531914091	0.122727272727273	0.189751178235797	0.0227272727272727	0.0454545454545455	930.302309513116	20.5325198890446	1.11360270941536E-05	3
H	3.5	fc.H_rm.3.5	0.852296865275761	0.781	0.0906514251086812	0.0860691265793709	0.814587713949958	0.106826128883306	0.50525	0.454680382246694	0.220454545454545	0.263727779344149	0.0227272727272727	0.0454545454545455	920.610171001967	10.8403813778957	0.00141693985316439	8
LQH	3.5	fc.LQH_rm.3.5	0.846708113534659	0.898	0.123448699428028	0.108468673401281	0.80057486069521	0.141112602979271	0.738	0.11008178777618	0.245454545454545	0.312018223667827	0.0477272727272727	0.0552355337380551	921.81376566087	12.0439760367997	0.000776236671504271	8
L	4	fc.L_rm.4	0.765802999881457	0.936	0.148399340056275	0.101409895306855	0.754457724287178	0.14717465142815	0.68475	0.210290869987263	0.147727272727273	0.238726534190738	0.0227272727272727	0.0454545454545455	937.582891726424	27.813102102353	2.92261721970122E-07	3
LQ	4	fc.LQ_rm.4	0.791375526035029	0.947	0.12576187948145	0.0833285219112665	0.783407954598038	0.125104911103819	0.70375	0.20438423781365	0.122727272727273	0.189751178235797	0.0227272727272727	0.0454545454545455	930.570904792485	20.8011151684141	9.73656023765993E-06	3
H	4	fc.H_rm.4	0.850836099339122	0.834	0.0931499640136725	0.0842353112051148	0.812289068299607	0.105959261320629	0.7225	0.083468556954101	0.195454545454545	0.282988771204971	0.0227272727272727	0.0454545454545455	916.246372530895	6.47658290682421	0.0125585128295754	6
LQH	4	fc.LQH_rm.4	0.842721916397475	0.91	0.128437298199748	0.108023126087222	0.799798653099077	0.14079722684296	0.77575	0.102844137736026	0.245454545454545	0.312018223667827	0.0227272727272727	0.0454545454545455	924.255196332847	14.4854067087761	0.000229004608257559	8
L	4.5	fc.L_rm.4.5	0.765904687638918	0.937	0.149008444359055	0.101614844137654	0.75369162148528	0.147360017263309	0.67725	0.209994245952915	0.147727272727273	0.238726534190738	0.0227272727272727	0.0454545454545455	937.797005392827	28.0272157687564	2.62589736182981E-07	3
LQ	4.5	fc.LQ_rm.4.5	0.79160372151261	0.944	0.127056835834976	0.0832575632186885	0.783036864833056	0.125593303974546	0.701	0.214253743646795	0.122727272727273	0.189751178235797	0.0227272727272727	0.0454545454545455	930.875241318114	21.1054516940427	8.36218397588701E-06	3
H	4.5	fc.H_rm.4.5	0.850508253563702	0.841	0.0919237071816671	0.088278498868609	0.805453336741385	0.104034641933083	0.72675	0.00956991814663707	0.195454545454545	0.282988771204971	0.0227272727272727	0.0454545454545455	915.195846705027	5.42605708095596	0.0212352314183776	5
LQH	4.5	fc.LQH_rm.4.5	0.840299932578609	0.9	0.13017684138013	0.105784414959846	0.799078789330839	0.139480995813717	0.79925	0.139437859516943	0.222727272727273	0.321369315452799	0.0227272727272727	0.0454545454545455	920.133744482469	10.3639548583984	0.0017980680320413	6
L	5	fc.L_rm.5	0.766030269241028	0.935	0.149578662075424	0.101856283483621	0.752842321712855	0.147496136988639	0.676	0.223284571791246	0.147727272727273	0.238726534190738	0.0227272727272727	0.0454545454545455	938.037874183866	28.2680845597949	2.32795056396111E-07	3
LQ	5	fc.LQ_rm.5	0.791795242717008	0.948	0.128498449238751	0.0831158798688363	0.782572497527714	0.126153853259266	0.7	0.23642052928345	0.122727272727273	0.189751178235797	0.0227272727272727	0.0454545454545455	931.215759797921	21.44597017385	7.05305174088633E-06	3
H	5	fc.H_rm.5	0.849905350739413	0.889	0.0951112284824276	0.0929844528545896	0.802612124333535	0.108121917765367	0.60375	0.238392358658298	0.197727272727273	0.271955980071075	0.0227272727272727	0.0454545454545455	917.028946788956	7.25915716488521	0.00849189024899297	5
LQH	5	fc.LQH_rm.5	0.839018555700442	0.908	0.131697645172513	0.103517691772147	0.798702224261208	0.136840546658138	0.78675	0.147122114811699	0.222727272727273	0.321369315452799	0.0227272727272727	0.0454545454545455	921.663098345074	11.893308721003	0.000836972421425115	6
